# Geographical and temporal differences in gastric and oesophageal cancer registration by subsite and morphology in Europe

**DOI:** 10.3389/fonc.2024.1250107

**Published:** 2024-02-20

**Authors:** Francesco Giusti, Carmen Martos, Manola Bettio, Raquel Negrão Carvalho, Manuel Zorzi, Stefano Guzzinati, Massimo Rugge

**Affiliations:** ^1^ European Commission, Joint Research Centre (JRC), Ispra, Italy; ^2^ Belgian Cancer Registry, Brussels, Belgium; ^3^ Regional Epidemiological Service Unit, Azienda Zero, Padova, Italy; ^4^ Veneto Tumor Registry, Azienda Zero, Padova, Italy; ^5^ Department of Medicine - DIMED, Surgical Pathology and Cytopathology Unit, Università degli Studi di Padova, Padova, Italy

**Keywords:** cancer registry, gastric cancer, Oesophageal cancer, data quality, Stomach/Oesophagus data quality

## Abstract

**Background:**

Gastric and oesophageal cancers pose a serious public health concern. In 2020 a total of 189,031 incident cases (136,038 stomach, 52,993 oesophagus) and 142,508 deaths (96,997 stomach, 45,511 oesophagus) were estimated in Europe. Oesophago-gastric cancers are a heterogeneous disease, with different aetiology and epidemiology for the various topographic subsites and main histopathological types. Topography subsite and morphology is key information to allow differentiating oesophago-gastric cancers. Correct registration and coding of such variables are fundamental in allowing proper description of the epidemiology of different subsites and histopathological types of oesophago-gastric cancers. The aim of this article is to highlight geographical and temporal variability in topography and morphology of oesophago-gastric cancers observed in Europe in the considered period.

**Methods:**

Data collected in the framework of the ENCR-JRC (European Commission’s Joint Research Centre) data call and feeding the European Cancer Information System (ECIS) were used to assess the variability of topography and morphology registration of gastric and oesophageal cancer in Europe in the period 1995-2014. Malignant cancers of the stomach and the oesophagus were selected following, respectively, topography codes C16 and C15 of the International Classification of Diseases for Oncology, third edition (ICD-O-3). Analyses were performed by subsite, morphology group, year, sex, and European region.

**Results:**

A total of 840,464 incident cases occurring in the period 1995-2014 – 579,264 gastric (67.2%) and 276,260 (32.8%) oesophageal carcinomas – was selected for the analysis. Data was recorded by 53 PBCRs (9 based in Northern Europe, 14 in Western Europe, 3 in Eastern Europe and 27 in Southern Europe) from 19 countries.

**Conclusion:**

A wide variability in oesophago-gastric cancers topographic subsites and histopathological types patterns was observed, with a corresponding improvement in accuracy of registration in the analysis period. PBCRs are ideally placed to guide the epidemiological evaluations of such a complex group of diseases, in collaboration with clinicians, patients and other public health stakeholders.

## 1 Introduction

Gastric and oesophageal cancers pose a serious public health concern. In 2020 a total of 189,031 incident cases (136,038 stomach, 52,993 oesophagus) and 142,508 deaths (96,997 stomach, 45,511 oesophagus) were estimated in Europe. Although incidence rates of gastric cancer have been decreasing in Europe since decades (1–4), due to ageing the burden of oesophago-gastric cancer is expected to further rise in absolute terms the in next decades, with an estimated 25% increase for gastric cancer (170,027 cases) and a 22% increase in oesophageal cancer (64,720 cases) by 2040 (5).

Oesophago-gastric cancers are a heterogeneous disease, with different aetiology and epidemiology for the various topographic subsites and main histopathological types. Stomach cancers can be classified in two anatomic sites, cardia gastric cancers (CGCs) (the upper part, next to the gastro-oesophageal junction) and non-cardia gastric cancers (NCGCs) (the lower part of the organ), and two main histological groups, diffuse and intestinal type. As for the stomach, oesophageal cancer are mainly adenocarcinomas; the two main histopathological subtypes are oesophageal squamous cell carcinoma (OSCC) and oesophageal adenocarcinoma (OAC) (6–8).

*Helicobacter pylori* infection is the most common cause of NCGCs, whereas it is not a known risk factor in CGC, and likely has a protective effect in OACs (9, 10). Tobacco smoking and alcohol consumption are the main risk factors for OSCCs, whereas Gastroesophageal reflux disease (GORD) and obesity are associated with OACs and CGCs (11, 12).

Population-based cancer registries (PBCRs) collect incidence data on all reportable neoplasms within a defined area. PBCRs have been in operation since the 1940s in a growing number of European countries, adhering to the international standards of the International Association of Cancer Registries (IACR) and the European Network of Cancer Registries (ENCR). PBCRs are currently evolving beyond their traditional role, adding critical information such as stage, treatment and biomarkers to their records. Among other purposes, such information can be also used to evaluate public health interventions, or inequalities in access to care (13–17).

Topography subsite and morphology is key information to allow differentiating oesophago-gastric cancers. Correct registration and coding of such variables is fundamental in allowing proper description of the epidemiology of different subsites and histopathological types of oesophago-gastric cancers.

This study aims to highlight geographical and temporal variability in topography and morphology of oesophago-gastric cancers observed in Europe in the period 1995-2013.

## 2 Methodology

Data collected in the framework of the ENCR-JRC (European Commission’s Joint Research Centre) data call (18) and feeding the European Cancer Information System (ECIS) (1) were used to assess the variability of topography and morphology registration of gastric and oesophageal cancer in Europe in the period 1995-2014, corresponding to the latest 20 years currently available in ECIS. In order to reduce fluctuations in data patterns, population-based PBCRs which submitted at least 17 out of 20 incidence years in the considered time interval were included in the study. Since the number of cases was smaller, figures do not show data for the year 2014.

Malignant cancers of the stomach and the oesophagus were selected following, respectively, topography codes C16 and C15 of the International Classification of Diseases for Oncology, third edition (ICD-O-3) (19). Carcinomas and unspecified types of cancer were considered for the study, whereas haematological malignancies (particularly primary lymphomas), gastrointestinal stromal tumours, sarcomas and neuroendocrine tumours were excluded. Carcinomas and unspecified types of cancer were 98.3% of all solid oesophago-gastric cancers. Tumours with a death certificate as only basis of diagnosis (representing 4.1% of solid tumours) were also excluded.

For the analyses by subsite, ICD-O-3 topography codes were grouped as follows:

- C15.0-C15.3, **Upper one-third of the oesophagus**.
- C15.4, **Middle one-third of the oesophagus**.
- C15.5, **Lower one-third of the oesophagus**.
- C15.8, **Overlapping lesion of the oesophagus**.
- C15.9, **Oesophagus, Not Otherwise Specified (NOS)**.
- C16.0, **CGC**.
- C16.1-C16.6 **NCGC**.
- C16.8, **Overlapping lesion of stomach**.
- C16.9, **Stomach, NOS**.

Morphology groups with corresponding ICD-O-3 codes for cancers of the oesophagus were:

- **NOS neoplasm or carcinoma** (8000-8005, 8010-8015, 8020-8022, 8050).
- **OAC** (8140-8149, 8160-8162, 8190-8221, 8260-8337, 8350-8551, 8570-8576, 8940-8941).
- **OSCC** (8052-8078, 8083-8084).

For stomach cancers, histological categories, based on the Laurén (7) classification, were:

- **NOS neoplasm or carcinoma** (8000-8005, 8010-8015, 8020-8022, 8050).
- Adenocarcinoma, **intestinal type** (8144), including mucinous adenocarcinoma (8480-8481) and tubular adenocarcinoma (8211).
- Carcinoma, **diffuse type** (8145), including signet-ring cell carcinoma (8490).
- **Adenocarcinoma, NOS** (8140).
- **Other specified carcinomas**.

**Figure 1:**
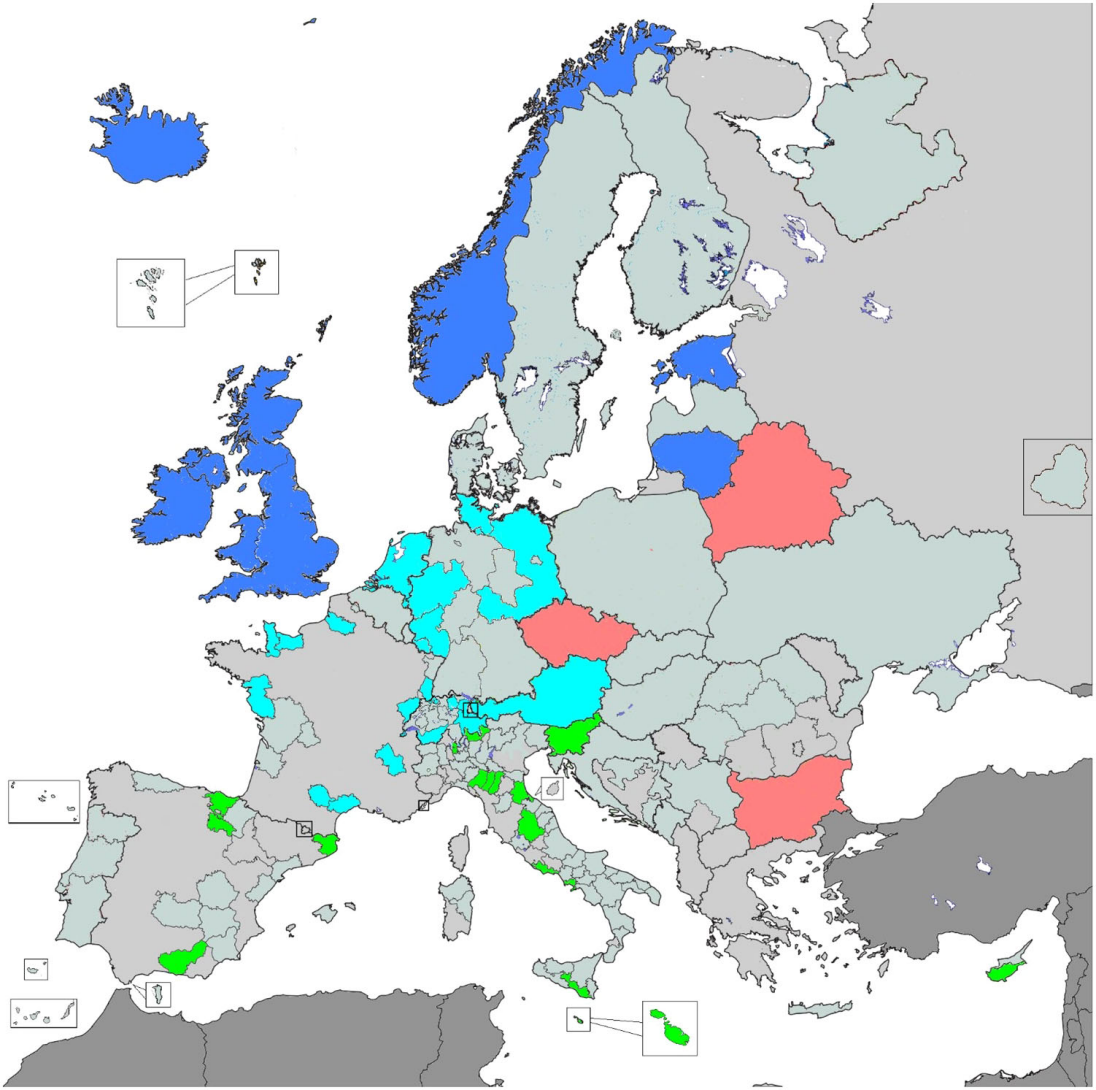
Selected PBCRs for the analysis, by European subregion. Blue corresponds to Northern Europe, cyan to Western Europe, red to Eastern Europe and green to Southern Europe (plus Cyprus).

For geographical comparison, Europe was considered as divided in regions (Northern Europe, Western Europe, Eastern Europe and Southern Europe) following the United Nations Statistics Division scheme (20) (see also **Figure 1**).

Age-standardised incidence rates were calculated by year, sex, topography, morphology and region considering the 2013 European Standard Population (21). Two-sided 95% confidence intervals were calculated using the normal approximation method and reported next to incidence rates.

The analysis was performed using the statistical software SAS Version 9.3 (SAS Institute Inc., Cary, NC, USA).

## 3 Results

A total of 840,464 incident cases occurring in the period 1995-2014 – 579,264 gastric (67.2%) and 276,260 (32.8%) oesophageal carcinomas – was selected for the analysis. Data was recorded by 53 PBCRs (9 based in Northern Europe, 14 in Western Europe, 3 in Eastern Europe and 27 in Southern Europe) from 19 countries (**Figure 1**).

Overall, cancer cases in males were 539,830 (64.2%) and 300,634 in females (35.8%). Carcinomas occurred in males and females respectively for 61.1% and 38.9% of gastric cases, and 70.7% and 29.3% of oesophageal cases. The median age at diagnosis was 73 [interquartile range (IQR) 64-78] for gastric cancer (72 [IQR 63-77] for males and 74 [IQR 67-80] for females), and 71 [IQR 62-77] for oesophageal cancer (69 [IQR 60-75] for males and 74 [IQR 66-82] for females).

### 3.1 Topography

#### 3.1.1 Overall temporal analysis

Oesophageal cancer incidence grew overall from 9.2 [9.0-9.4] cases per 100,000 in 1995 to 10.5 [10.3-10.7] cases in 2013 (**Supplementary Figure 1**). An increase in incidence was observed in Northern Europe (from 12.6 [12.3-13.0] to 14.5 [14.1-14.9] cases per 100,000), Western Europe (from 6.9 [6.4-7.4] to 9.5 [9.1-9.8]) and Eastern Europe (from 3.5 [3.1-3.9] to 5.6 [5.2-6.0]), whereas oesophageal cancer incidence decreased in Southern Europe (from 7.1 [6.5-7.8] to 5.9 [5.2-6.5]) between 1995 and 2013 respectively (**Supplementary Figure 2**).

**Figure 2:**
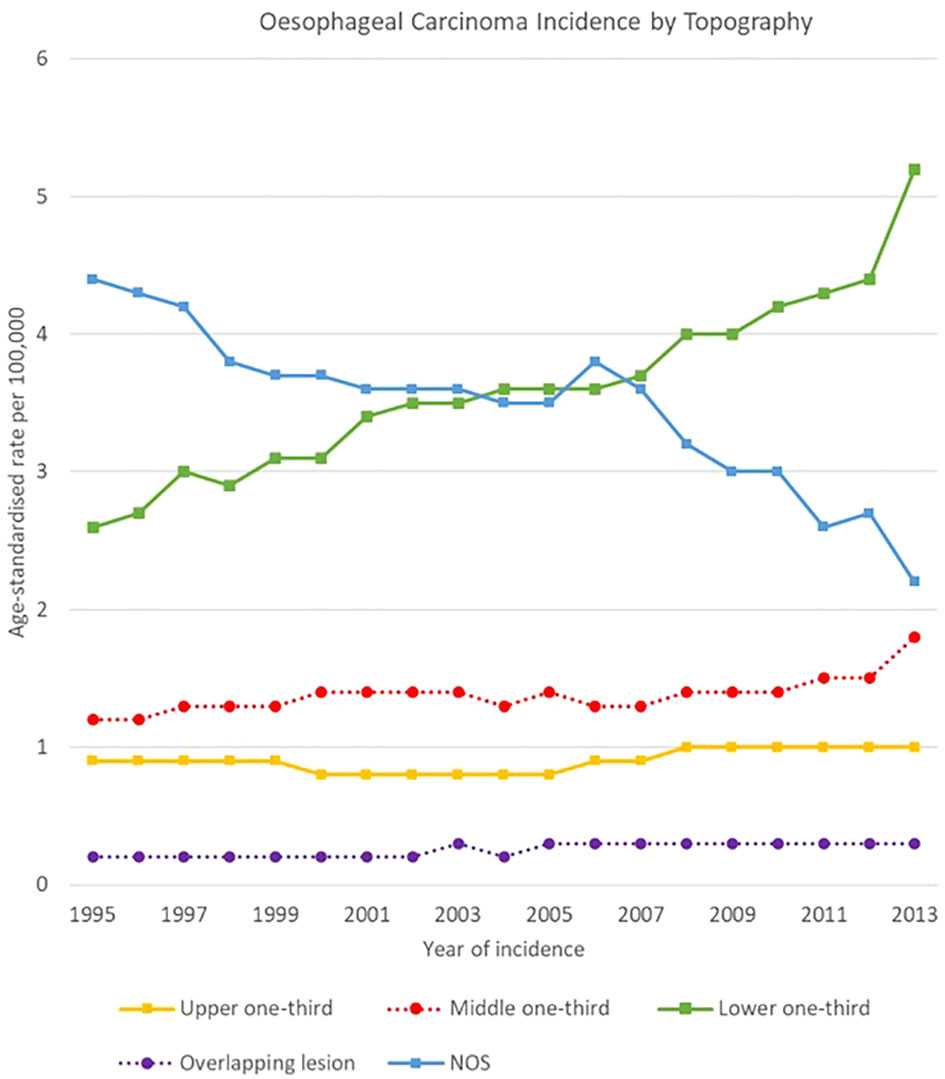
Time trends in oesophageal carcinoma incidence by topography, 1995-2013.

Analysis by anatomical subsite showed that incidence of carcinomas in the upper third of the oesophagus remained stable in the study period (0.9 [0.8-0.9] cases per 100,000 in 1995 – 1.0 [1.0-1.1] cases in 2013), whereas incidence in the middle third increased from 1.2 [1.1-1.2] to 1.8 [1.7-1.9] in the same period (**Figure 2**). Incidence of carcinomas to the lower third of the oesophagus rose from 2.6 [2.5-2.7] cases per 100,000 in 1995 to 5.2 [5.1-5.4] in 2013, showing opposite trends as compared to NOS cancers (likely to arise for the majority from the lower third), which decreased from 4.4 [4.3-4.6] to 2.2 [2.1-2.3] cases per 100,000 in the same period. Overall, the sum of lower third and NOS oesophageal cancer rates grew from 7.0 [6.9-7.1] cases per 100,000 in 1995 to 7.4 [7.3-7.5] in 2013 (**Figure 2**). Overlapping lesion was the subsite with the lowest incidence, with rates between 0.2 [0.2-0.2] and 0.3 [0.3-0.4] cases per 100,000 in 1995-2013.

An overall decline of gastric cancer incidence was observed in the period 1995-2013, with rates decreasing from 25.0 [24.6-25.3] to 15.0 [14.8-15.2] cases per 100,000 (**Supplementary Figure 3**). The decrease in incidence was observed in all European regions: Northern Europe (from 22.5 [22.0-23.0] to 11.4 [11.1-11.7] cases per 100,000), Western Europe (from 21.9 [21.2-22.6] to 14.4 [14.0-14.8]), Eastern Europe (from 31.2 [30.3-32.1] to 22.9 [22.1-23.7]) and Southern Europe (from 28.7 [27.5-29.8] to 15.7 [14.9-16.6]) between 1995 and 2013 respectively (**Supplementary Figure 4**).

**Figure 3:**
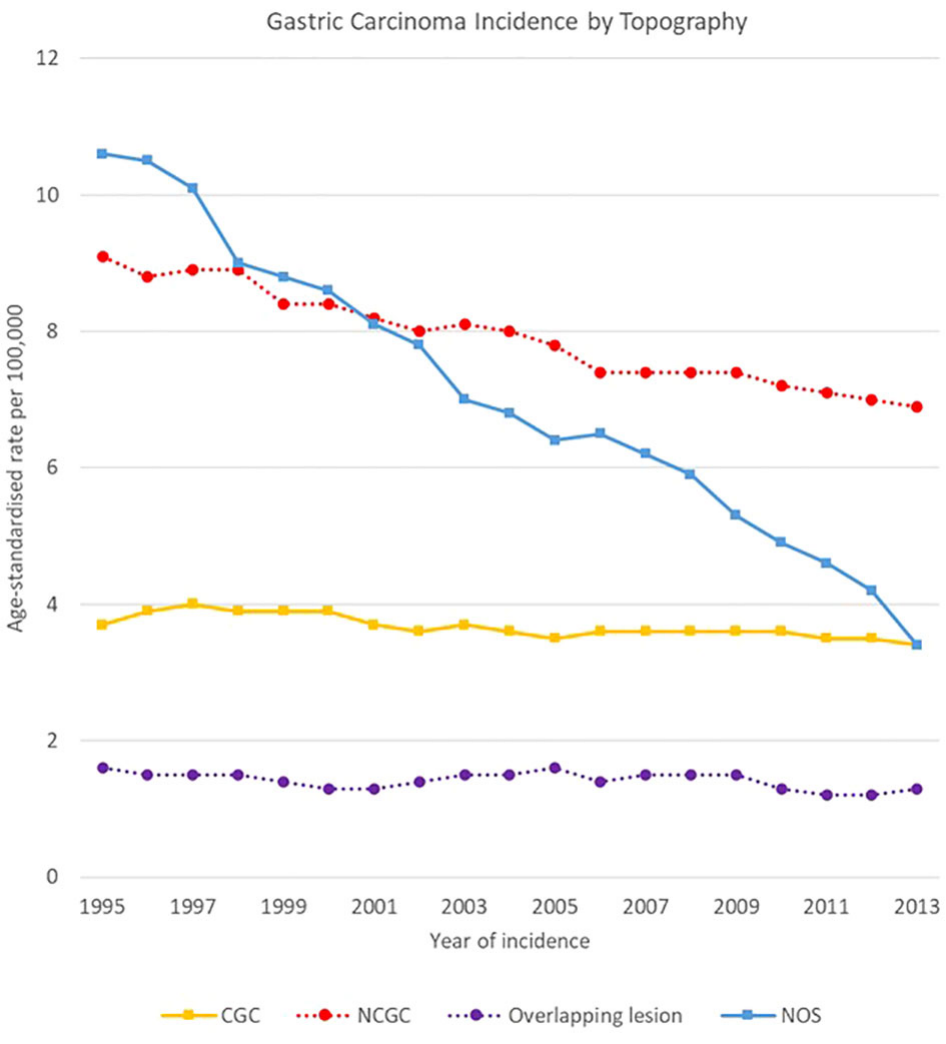
Time trends in gastric carcinoma incidence by topography, 1995-2013.

The decreasing trend was due to the NOS subsite of stomach decline, which fell from 10,6 [10.3-10.8] to 3,4 [3.3-3.6] cases per 100,000, as well as to NCGCs, decreasing from 9.1 [8.9-9.3] to 6.9 [6.8-7.1] cases per 100,000. Rates for CGCs and overlapping lesions of the stomach were more stable, with respectively a mean rate of 3.7 [3.5-3.8] and 1.4 [1.4-1.5] cases per 100,000 in the analysis period (**Figure 3**).

#### 3.1.2 Temporal analysis by geographical area

High variability was observed in the analysis by region, subsite and sex in oesophago-gastric carcinomas.

NCGC declined in all European regions, both for men and women between 1995 and 2013. For males, NCGCs had higher incidence rates than oesophageal carcinomas throughout all the analysis period in Southern and Eastern Europe, whereas oesophageal carcinomas took over NCGCs as the most incident oesophago-gastric cancer in 2002 in Northern Europe and in 2013 in Western Europe. Notably, NCGC rates remained high in Eastern Europe for both sexes, decreasing from 39.2 [37.4-40.9] to 29.2 [27.8-30.6] cases per 100,000 and from 20.0 [19.1-20.9] cases to 14.0 [13.2-14.8] cases per 100,000 for men and women respectively.

**Figure 4:**
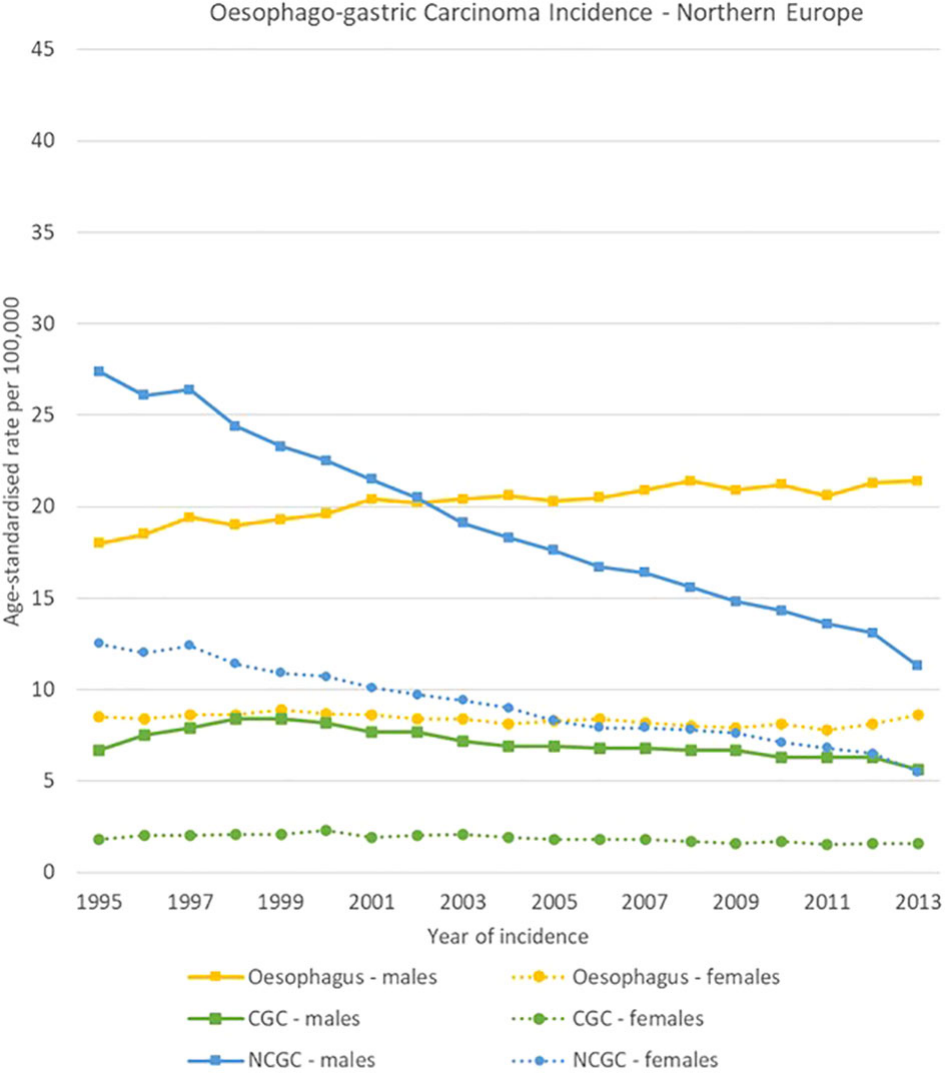
Time trends for oesophago-gastric carcinomas by topography and sex in Northern Europe, 1995-2013.

**Figure 5:**
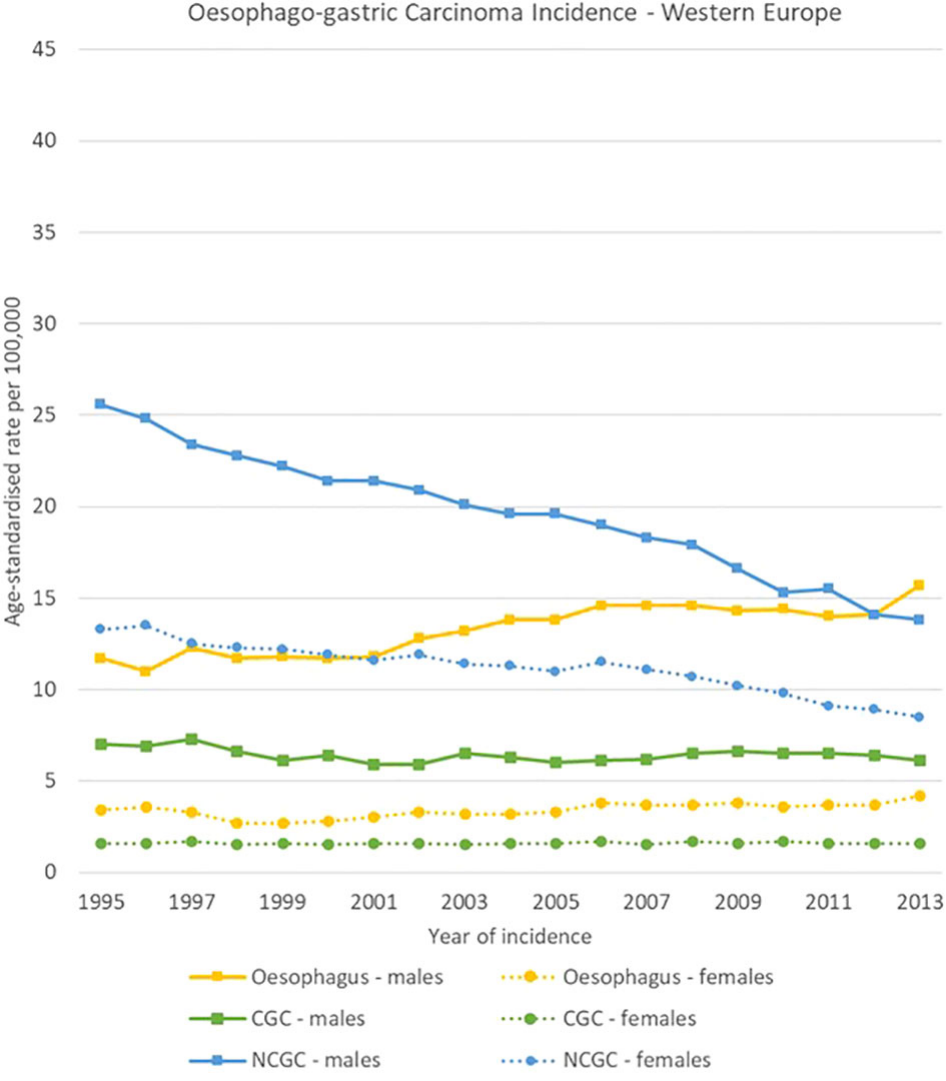
Time trends for oesophago-gastric carcinomas by topography and sex in Western Europe, 1995-2013.

**Figure 6:**
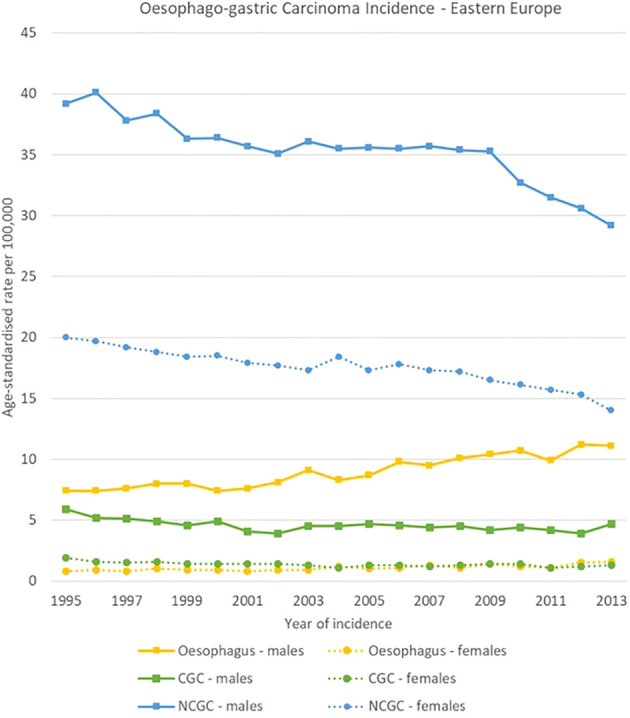
Time trends in oesophago-gastric carcinomas by topography and sex in Eastern Europe, 1995-2013.

**Figure 7:**
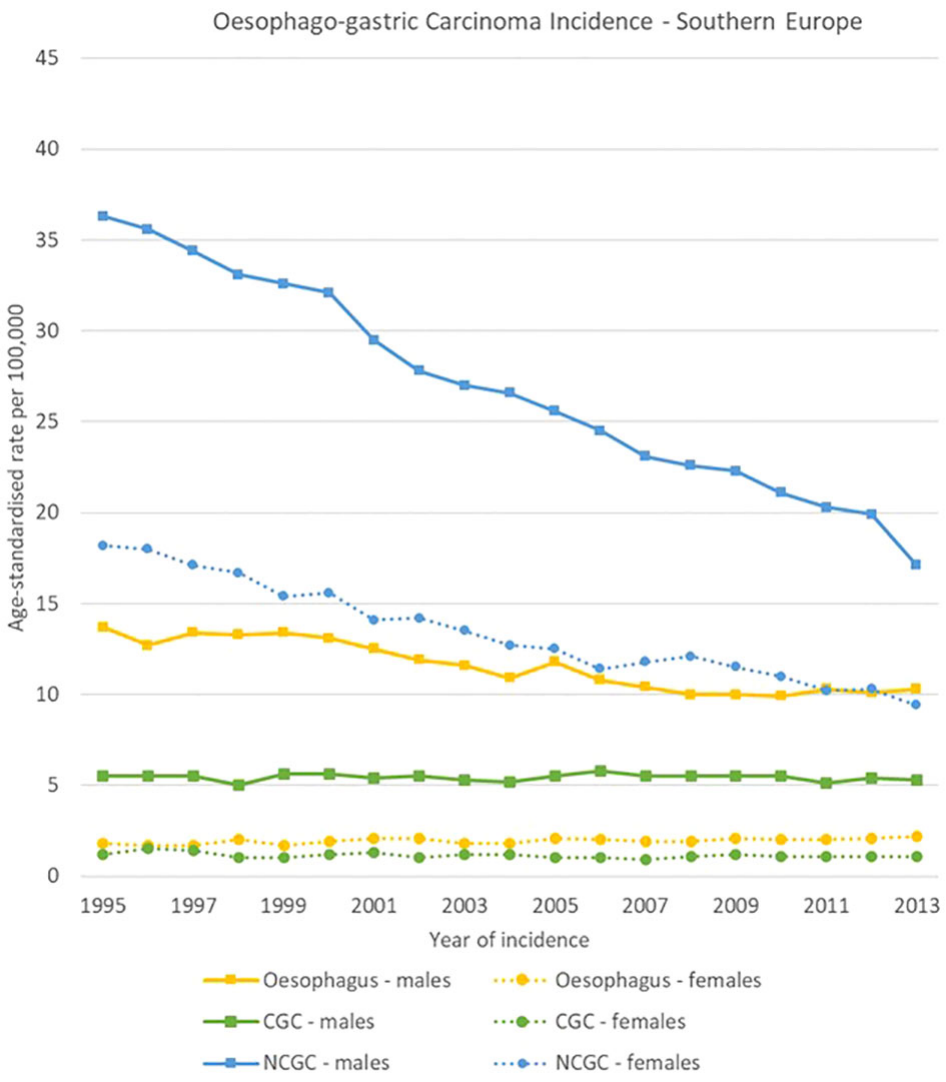
Time trends in oesophago-gastric carcinomas by topography and sex in Southern Europe, 1995-2013.

CGC rates were stable between 1995 and 2013 in all regions and for both sexes, with around 5-7 cases per 100,000 for men and around 1-2 cases per 100,000 for women (**Figures 4**–**7**).

### 3.2 Morphology

#### 3.2.1 Oesophageal carcinomas

The geographical comparison of oesophageal carcinomas morphology groups showed rather different trends and ratios for males across the four European regions, while less variability occurred in females.

In Northern Europe, OSCC rates were stable (from 4.9 [4.4-5.3] to 4.8 [4.3-5.2] cases per 100,000), while OAC rates increased from 9.0 [8.5-9.6] to 15.0 [14.4-15.7] cases per 100,000. The ratio between OSCCs and OACs rose from 1 to 1.8 in 1995 to 1 to 3.1 in 2013. In Western Europe, OSCCs rates were also stable (from 5.8 [5.2-6.5] to 5.9 [5.4-6.3] cases per 100,000), and OACs increased (from 4.6 [4.0-5.2] to 8.8 [8.2-9.4] cases per 100,000). In Eastern Europe, incidence rates for both OACs and OSCCs increased between 1995 and 2013 from 1.1 [0.7-1.6] to 3.0 [2.4-3.5] cases and from 3.7 [3.0-4.4] to 7.1 [6.3-8.0] cases per 100,000 respectively; OACs were less common than OSCCs, with a ratio of 1 to 3.4 in 1995 and 1 to 2.4 in 2013. In Southern Europe, a sharp decline in OSCCs incidence was observed from 10.5 [9.2-11.8] to 5.8 [4.9-6.7] cases per 100,000, while OACs rates went from 1.9 [1.2-2.5] to 4.1 [3.3-4.8] cases per 100,000.

**Figure 8:**
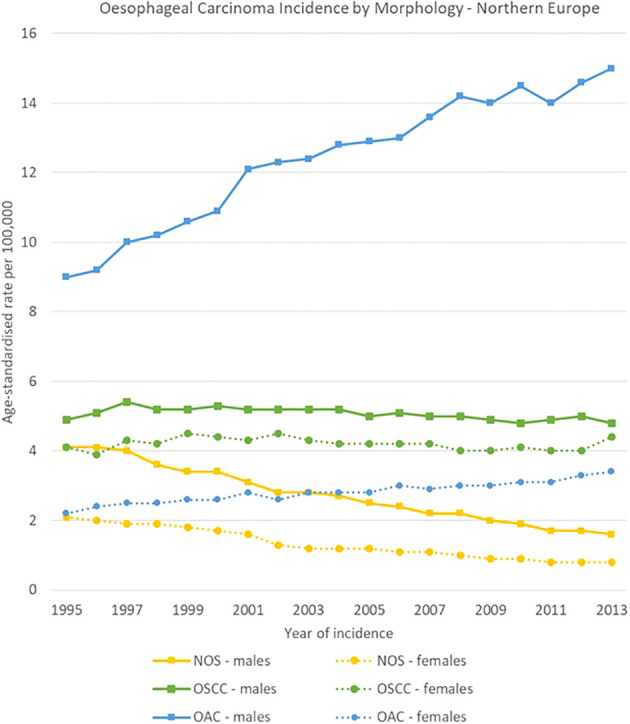
Time trends in oesophageal carcinomas by morphology and sex in Northern Europe, 1995-2013.

**Figure 9:**
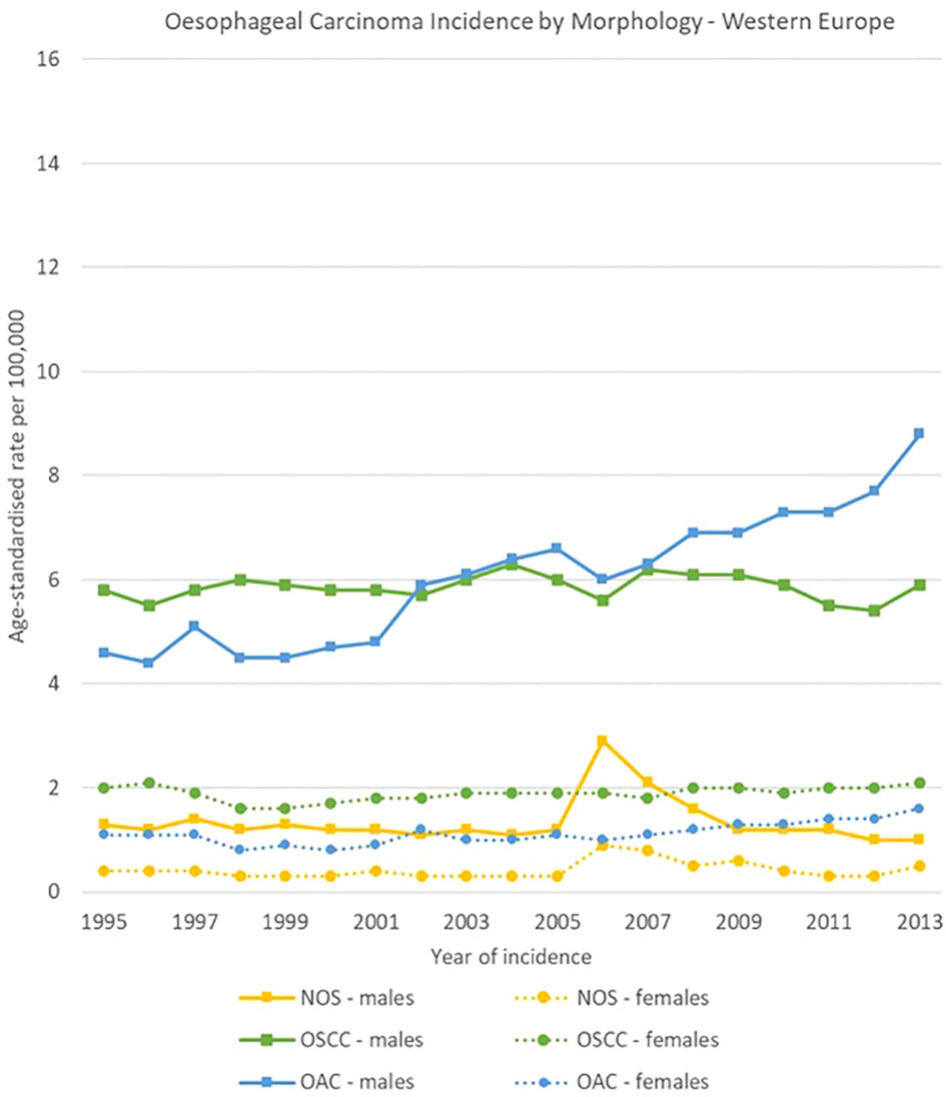
Time trends in oesophageal carcinomas by morphology and sex in Western Europe, 1995-2013.

**Figure 10:**
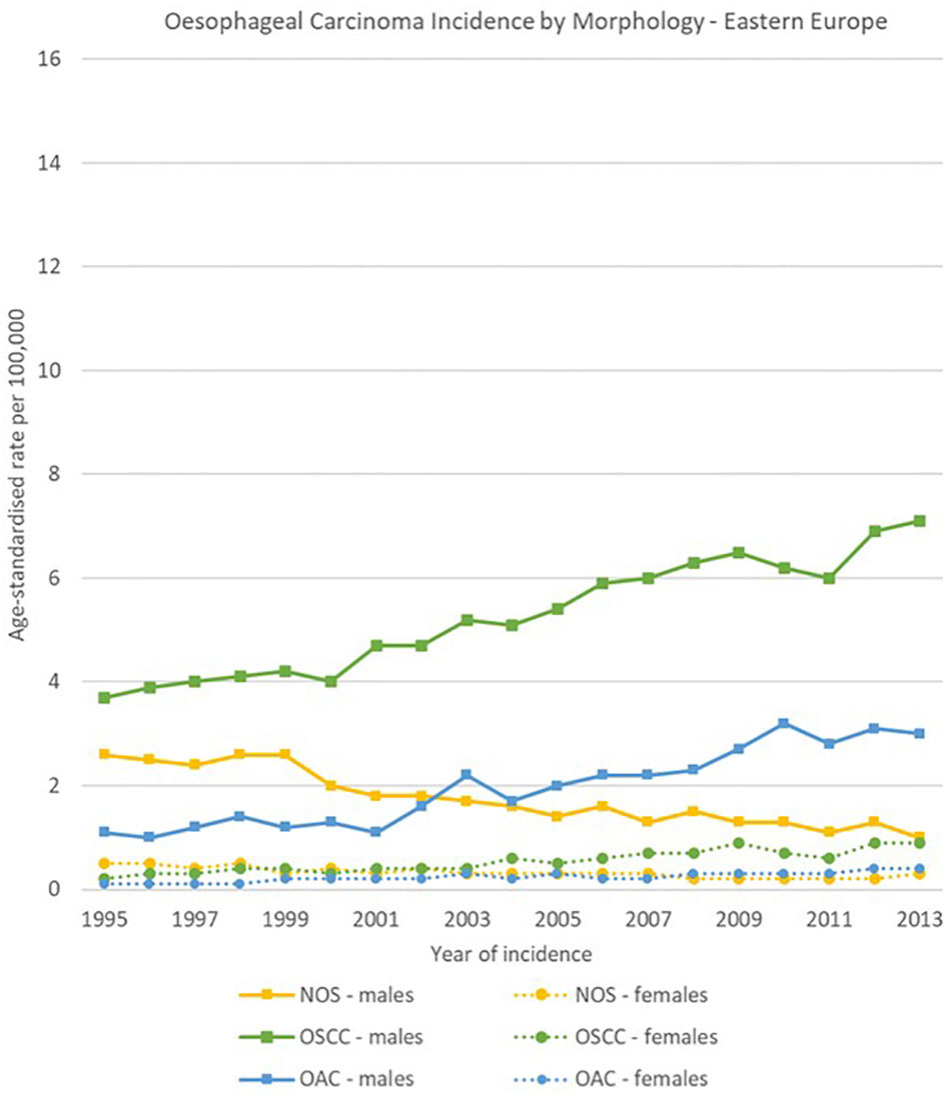
Time trends in oesophageal carcinomas by morphology and sex in Eastern Europe, 1995-2013.

**Figure 11:**
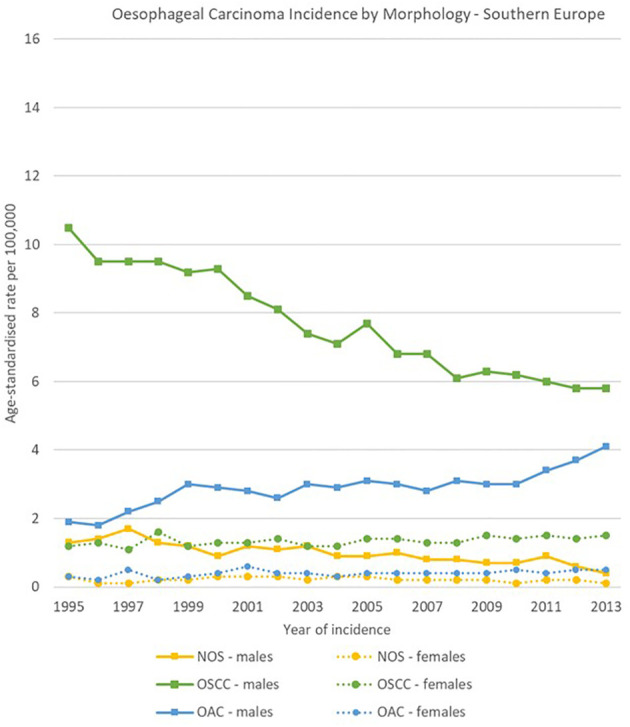
Time trends in oesophageal carcinomas by morphology and sex in Southern Europe, 1995-2013.

For females, incidence of OSCCs was higher than for OACs consistently in the four European subregions and for all the period 1995-2013. Among morphology groups, in 2013 the higher incidence in OSCC occurred in Northern Europe with 4.4 [4.0-4.8] cases per 100,000, and the lowest in Eastern Europe with 0.9 [0.7-1.2] cases per 100,000 (**Figures 8**–**11**).

#### 3.2.2 Gastric carcinomas

Morphology groups of stomach carcinomas also showed different trends in the analysis period. Incidence rates generally decreased in the two less specific groups (adenocarcinomas NOS and NOS neoplasms or carcinomas) and were stable instead for most of the other groups. In particular, NOS neoplasms or carcinomas decreased sharply in Eastern Europe, from 12.5 [11.9-13.0] cases per 100,000 in 1995 to 2.5 [2.3-2.8] cases per 100,000 in 2013, and adenocarcinomas NOS incidence fell from 11.8 [11.0-12.6] to 4.9 [4.4-5.4] cases per 100,000 in Southern Europe.

**Figure 12:**
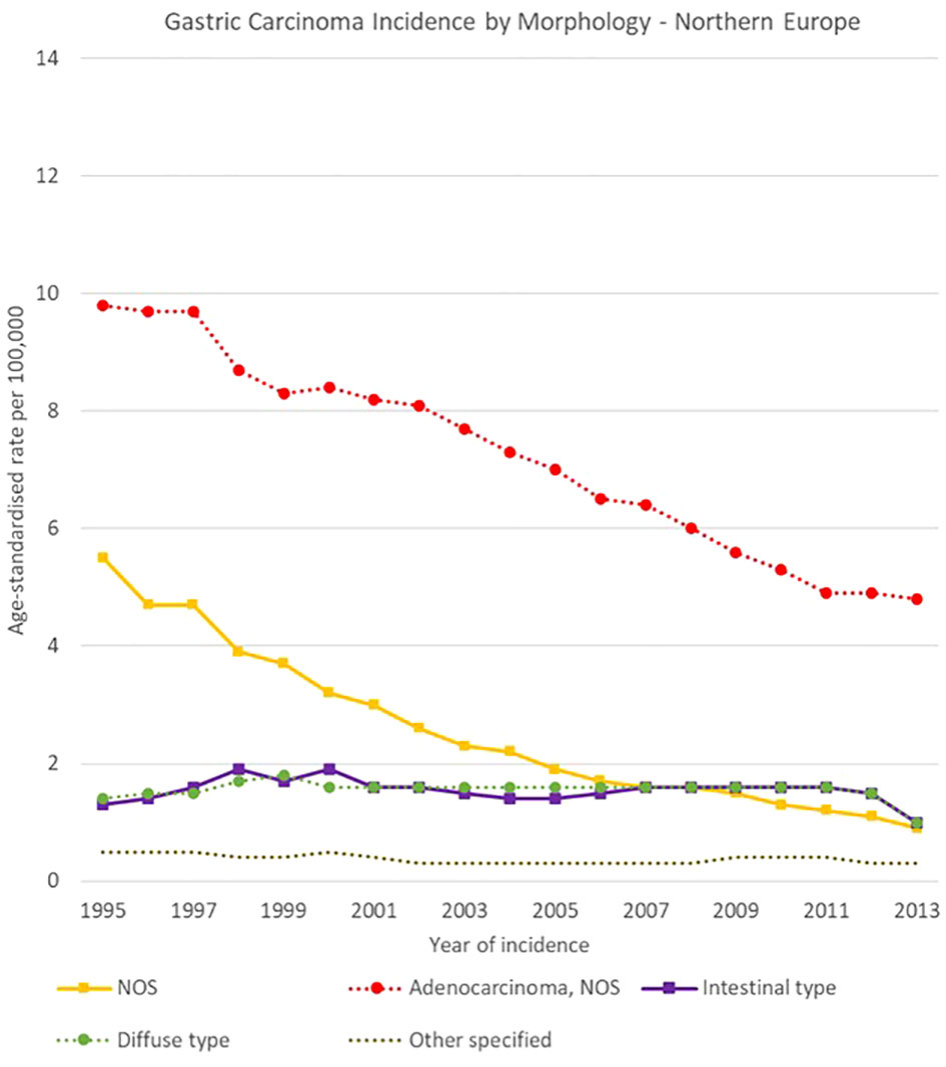
Time trends in gastric carcinomas by morphology in Northern Europe, 1995-2013.

**Figure 13:**
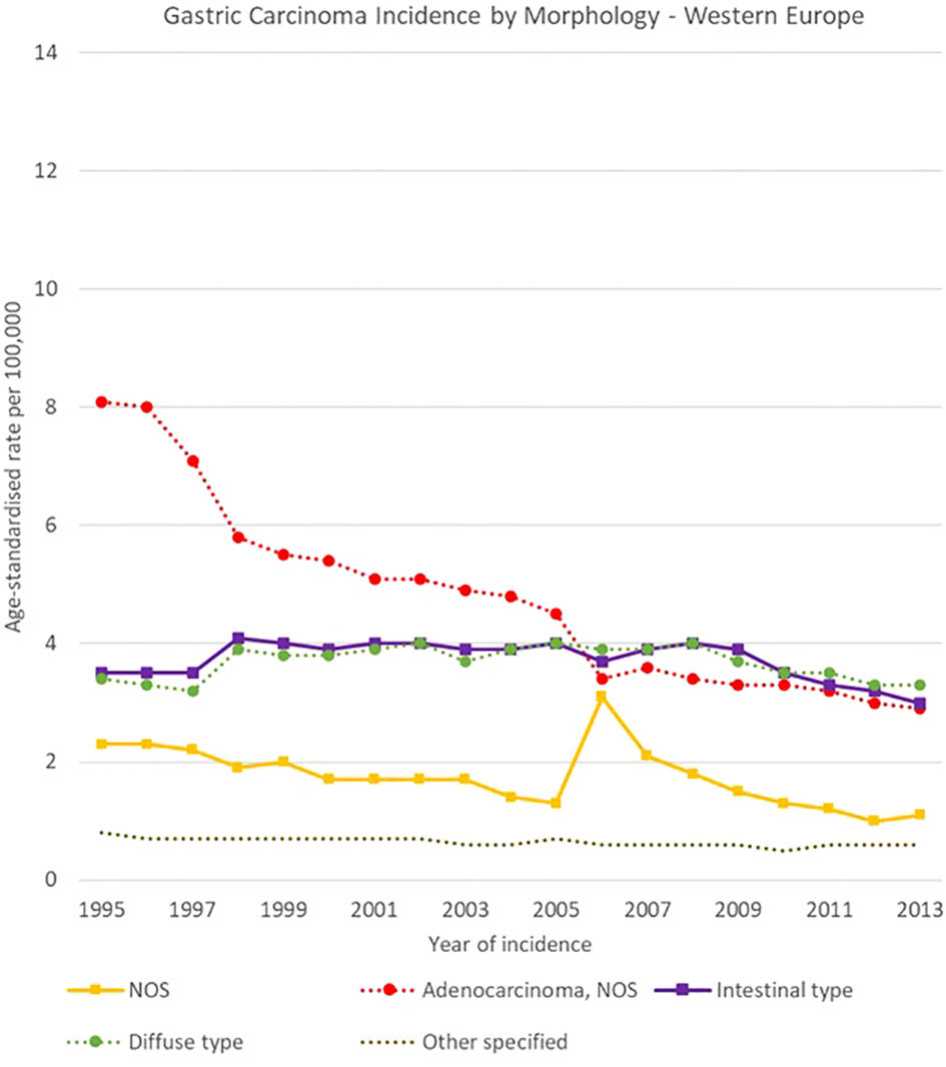
Time trends in gastric carcinomas by morphology in Western Europe, 1995-2013.

**Figure 14:**
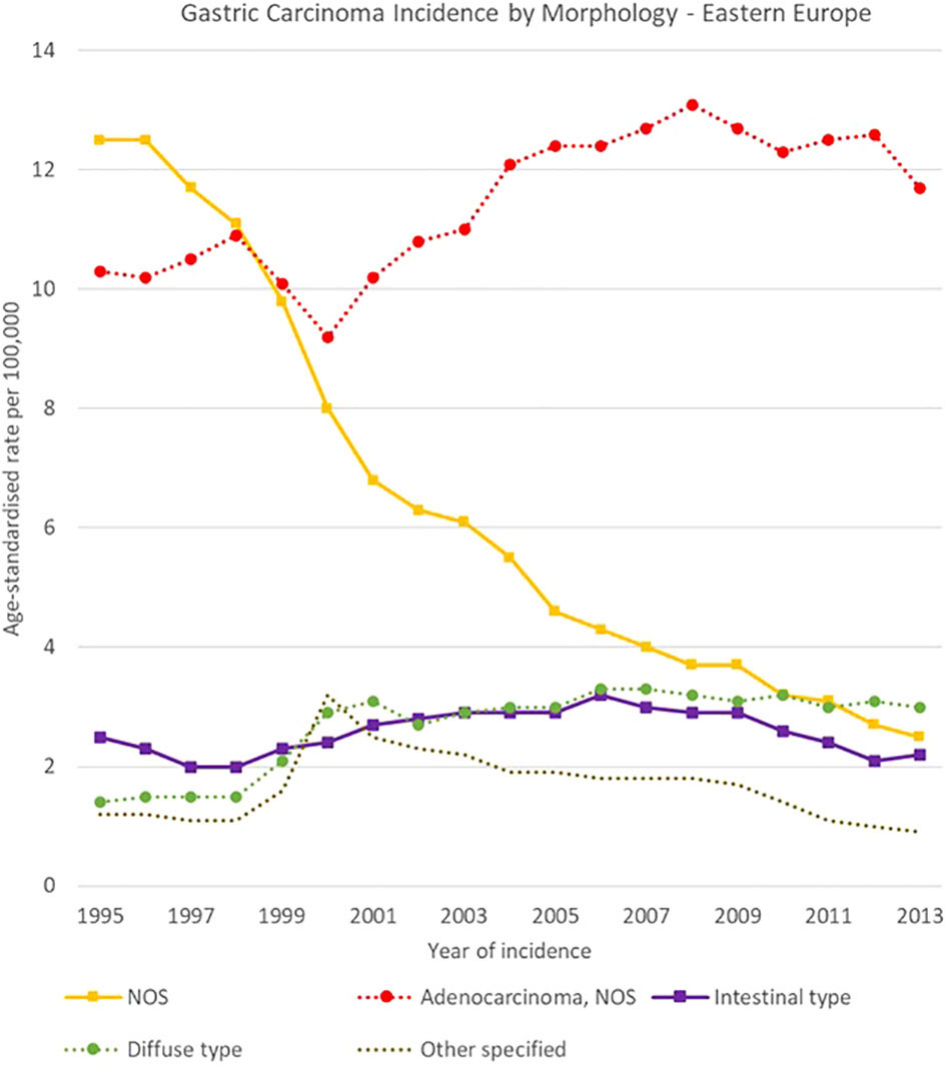
Time trends in gastric carcinomas by morphology in Eastern Europe, 1995-2013.

**Figure 15:**
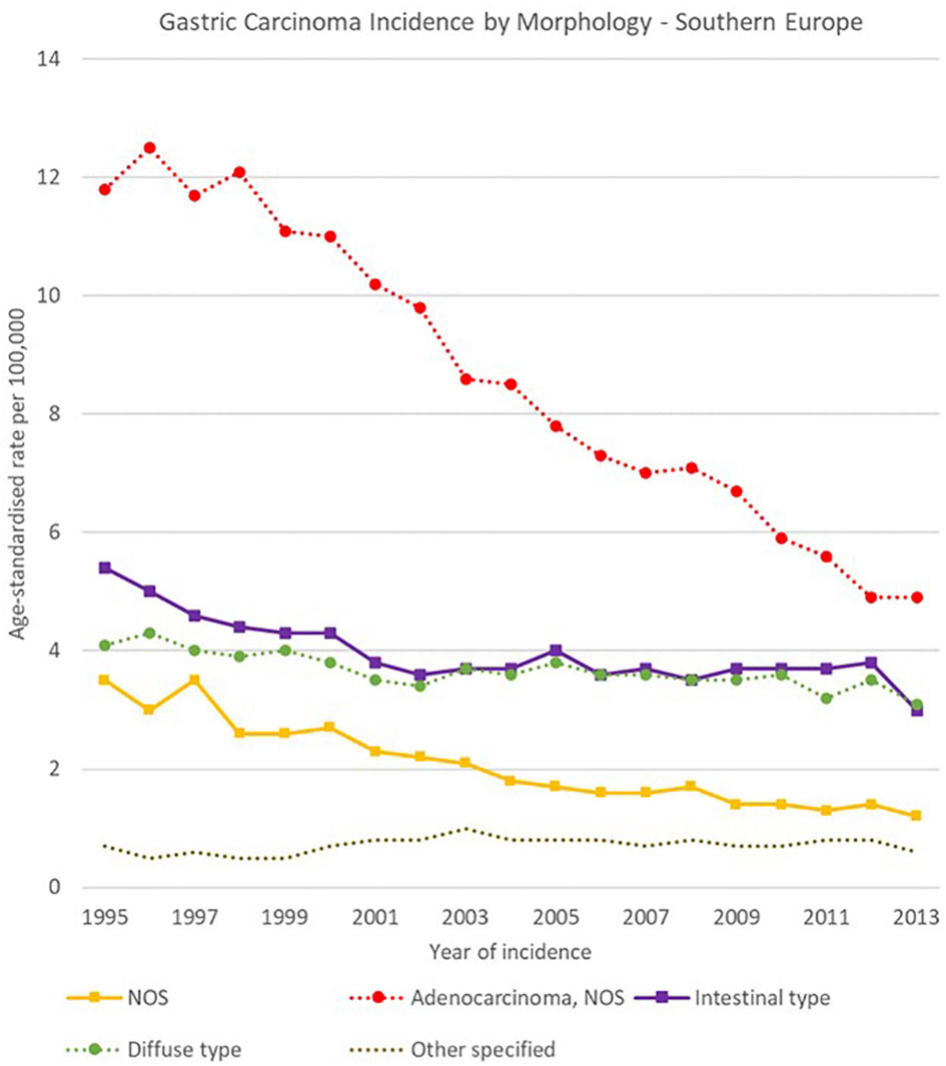
Time trends in gastric carcinomas by morphology in Southern Europe, 1995-2013.

Specified morphology groups also showed variability among European regions. For instance, intestinal-type adenocarcinomas incidence changed from 5.4 [4.9-6.0] to 3.0 [2.5-3.4] cases per 100,000 in Southern Europe, and from 1.3 [1.2-1.4] to 1.0 [0.9-1.2] cases per 100,000 in Northern Europe. Higher incidence occurred for diffuse type carcinomas in Southern Europe, where it decreased from 4.1 [3.7-4.6] to 3.1 [2.7-3.5] cases per 100,000 in 1995-2013 respectively. Incidence rates in Northern Europe instead decreased from 1.4 [1.3-1.6] to 1.0 [0.9-1.1] cases per 100,000 in the same period (**Figures 12**–**15**).

## 4 Discussion

To our best knowledge, this is the first analysis addressing long-term changes in topography and morphology registration of both gastric and oesophageal cancers in Europe by area and sex.

Within upper gastrointestinal tract, cancer topography, combined with histological subtyping, have crucial impact on cancer prevention strategies, diagnostic procedures, the best choices of treatments and, ultimately, in the estimates of prognosis. Unfortunately, these important data are unequally provided by non-high-resolution cancer registration, which results in fragmentary, and sometimes divergent, epidemiological information.

Among the main strengths of the study are the large number of the selected cases (840,464 oesophago-gastric cancers), the length of the analysis period (1995-2014) and the geographical representation (53 PBCRs from Northern, Western, Eastern and Southern Europe).

In order to assess the representativity of data by European region, checks on incidence rates for gastric and oesophageal cancer in areas for which registration could not cover at least 17 years in the analysis period were performed. The incidence of gastric and oesophageal cancers was checked for all PBCRs with data available in 1995 and in 2013, and the rates were close overall to those reported in the study.

As a possible limitation, variations in data collection and coding practices could partly limit the generalisability of the study, even though a constant improvement in data quality has been reported for European PBCRs in the same period (22).

### 4.1 Different epidemiological patterns of cancer incidence across Europe

The present study revealed notable differences in the reporting and registration of upper gastrointestinal cancers across Europe, over time. Overall, it was found that there was an improvement in identifying specific subsites of oesophageal and gastric cancers between 1995 and 2013.

As for the oesophagus, there was a rising incidence of cancer in the lower one-third of the oesophageal channel, while the registration of malignancies with unspecified histology showed a decrease. Other subsites, however, did not exhibit significant changes.

Between 1995 and 2014, OAC increased consistently across European regions, in line with what already reported (6, 23–25). However, the extent of this increase varied by region and gender. Females, in particular, had higher incidence rates in Northern Europe than in Southern and Eastern Europe. These differences could be attributed to the varying effects of cancer-promoting factors such as gastro-oesophageal reflux, obesity, and low *Helicobacter pylori* prevalence in Northern European countries (12, 26–28) versus a cancer-protective effect observed in Southern and Eastern European populations due to higher rates of *Helicobacter pylori* infection (9, 10, 29).

The occurrence of OSCC varied among European regions. OSCC decreased only in males of Southern Europe, remained steady in Northern and Western Europe, and increased in both sexes in Eastern Europe. These differences could be attributed to the greater effects of tobacco smoking and alcohol consumption among Eastern European populations (11, 30).

Consistently with the current literature, gastric cancer incidence decreased dramatically (10, 23, 31, 32). In particular, NCGC rates became lower than those of oesophageal cancer in 2003 in Northern Europe, and in 2013 in Western Europe. The variability in gastric/oesophageal cancers ratio between European areas likely results from the differences in the prevalence of the different risk factors, particularly *Helicobacter pylori* infection (29).

Such evidence should prompt the implementation of internationally validated strategies for gastric cancer prevention (primary and secondary) (33, 34).

The decrease in NOS subsite proportion of gastric cancer coupled with stable rates of cardia-gastric adenocarcinoma. This was evidenced by the CGC : NCGC ratio, which remained steady from 1995 (1:2.5) to 2013 (1:2.1).

### 4.2 Data accuracy of cancer registration

#### 4.2.1 Oesophageal and gastroesophageal-junction malignancies

Between 1995 and 2014, the quality of registration of oesophageal cancer improved significantly across Europe despite some variations among the regions. The incidence of malignancies with either unspecified subsite and/or morphology reduced overall across Europe.

Notably, in Northern European countries, the incidence of malignancies with unspecified morphology dropped from 4.1 to 1.6 cases per 100,000 in males, and specific cancer morphological groups such as oesophageal adenocarcinoma became more frequently identified, with an increase in incidence from 9.0 to 15.1 cases per 100,000. However, the incidence of OSCC did not show any significant change during this period for both sexes.

These findings underscore the importance of reducing the proportion of oesophageal cancers with unspecified morphology, given the different treatment strategies for OAC and OSCC (6, 35–39).

The improvement in diagnostic tools, such as high magnification and ultrasound endoscopy, played a significant role in promoting cancer histological subtyping (40). Along with that, the advent of digital pathology databases has helped to ensure consistent delivery of histological information from local pathology archives to centralised cancer registrations. Lastly, the HER-2 revolution has played a crucial role in the development of cancer-personalised therapies, making molecular profiling of oesophageal adenocarcinoma mandatory (38, 41).

#### 4.2.2 Gastric malignancies

A strong decrease in the proportion of stomach NOS subsite was observed, with a decline in incidence from 10,6 to 3,4 cases per 100,000 between 1995 and 2013. The increase in the accuracy of registration of gastric cancers either as CGCs or NCGCs is crucial, given the different clinical characteristics and outcomes between the two subsites (6, 35, 37, 42, 43).

In addition, due to the differences (such as clinical features, genetics, surgery) between intestinal and diffuse type gastric cancers it is important for PBCRs to record the morphology of incident cancers with the best possible accuracy (44, 45).

With the only notable exception of Eastern Europe, the quality improvement in gastric cancer registration showed a trend even more favourable than that of oesophageal malignancies. Moreover, the drop in the incidence of NOS adenocarcinoma histotypes and NOS malignancies mirrors the lowering incidence of primary gastric epithelial malignancies as consistently documented (with the abovementioned exception) all over the considered European regions. This situation is consistent with the regional prevalence of the leading risk factor of gastric adenocarcinoma (*Helicobacter pylori*, with exceedingly higher prevalence in Eastern Europe) (29).

## 5 Conclusion and way forward

A wide variability in oesophago-gastric cancers topographic subsites and histopathological types patterns was observed, with a corresponding improvement in accuracy of registration in the analysis period. PBCRs are ideally placed to guide the epidemiological evaluations of such a complex group of diseases, in collaboration with clinicians, patients and other public health stakeholders.

JRC and ENCR have been supporting high level of quality and the harmonisation of European population-level cancer incidence data, though several initiatives such as drafting of recommendations for PBCR data coding, the organisation of trainings for PBCR personnel, and the developments of IT tools for data quality checks (46–50).

In the context of such activities, the present analysis on geographical and temporal differences in gastric and oesophageal cancer registration is a first step towards a more in-depth evaluation of the burden of these diseases in Europe.

## Data availability statement

The original contributions presented in the study are included in the article/**Supplementary Material**, further inquiries can be directed to the corresponding author/s.

## Author contributions

The first draft of the manuscript was written by FG, CM and MR. All authors contributed to the article and approved the submitted version.
