# Supplementary figures and images for: Geographical and temporal differences in gastric and oesophageal cancer registration by subsite and morphology in Europe
Source: Front Oncol. 2024 Feb 20;14:1250107. doi: 10.3389/fonc.2024.1250107 (PMC10912620; doi:10.3389/fonc.2024.1250107)

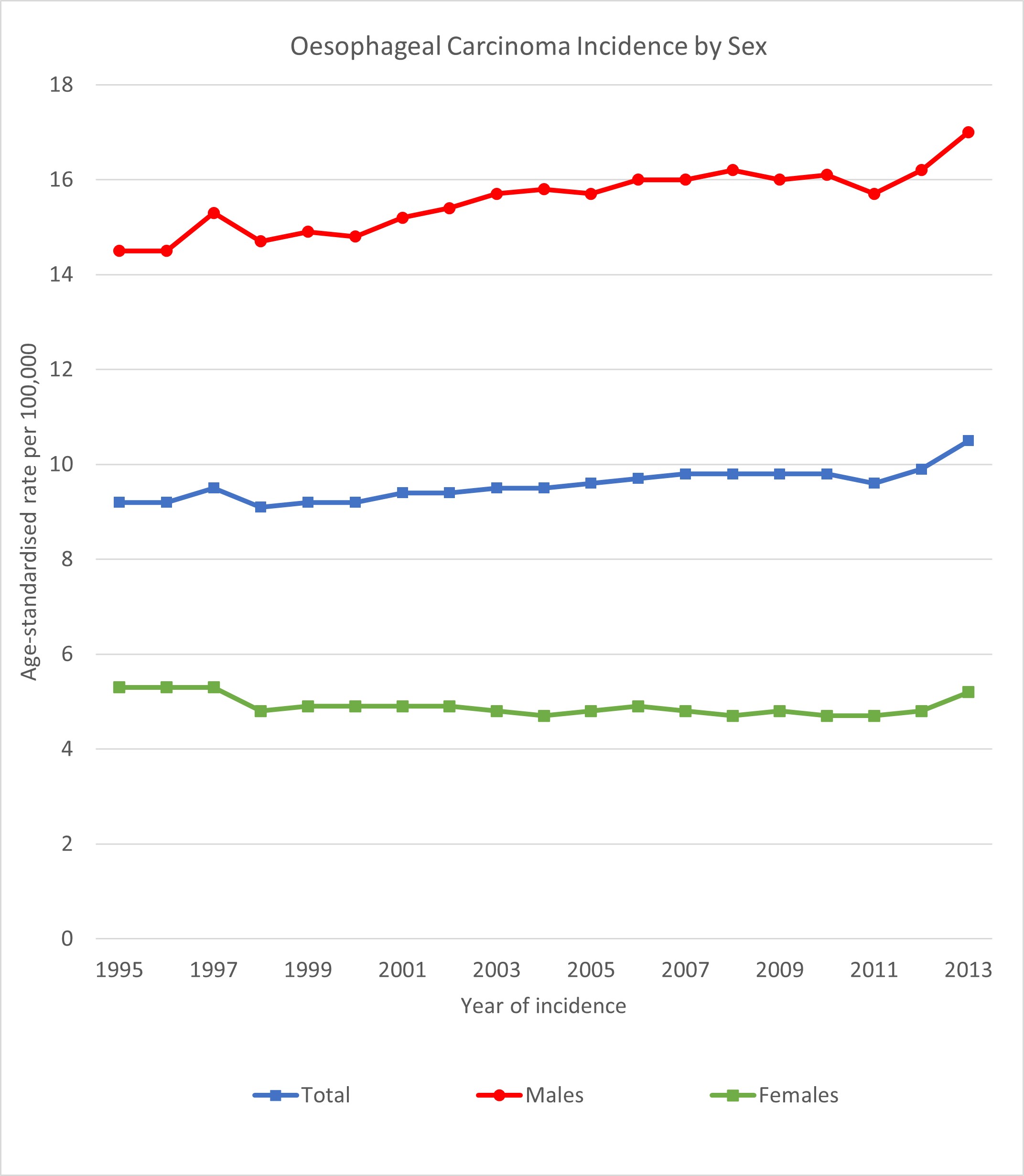

Supplement: Supplementary file 1 [file Image_1.jpeg]

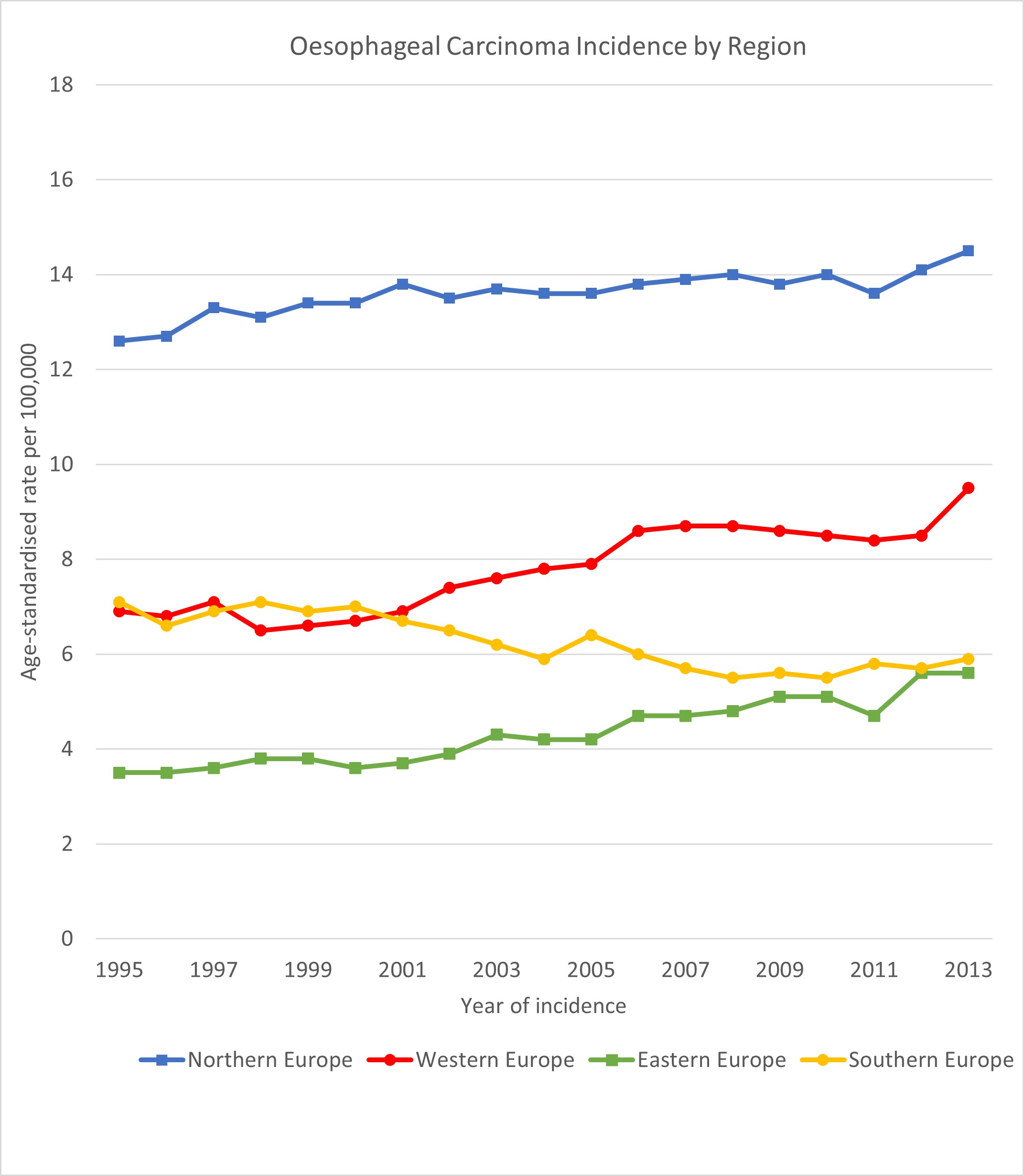

Supplement: Supplementary file 2 [file Image_2.jpeg]

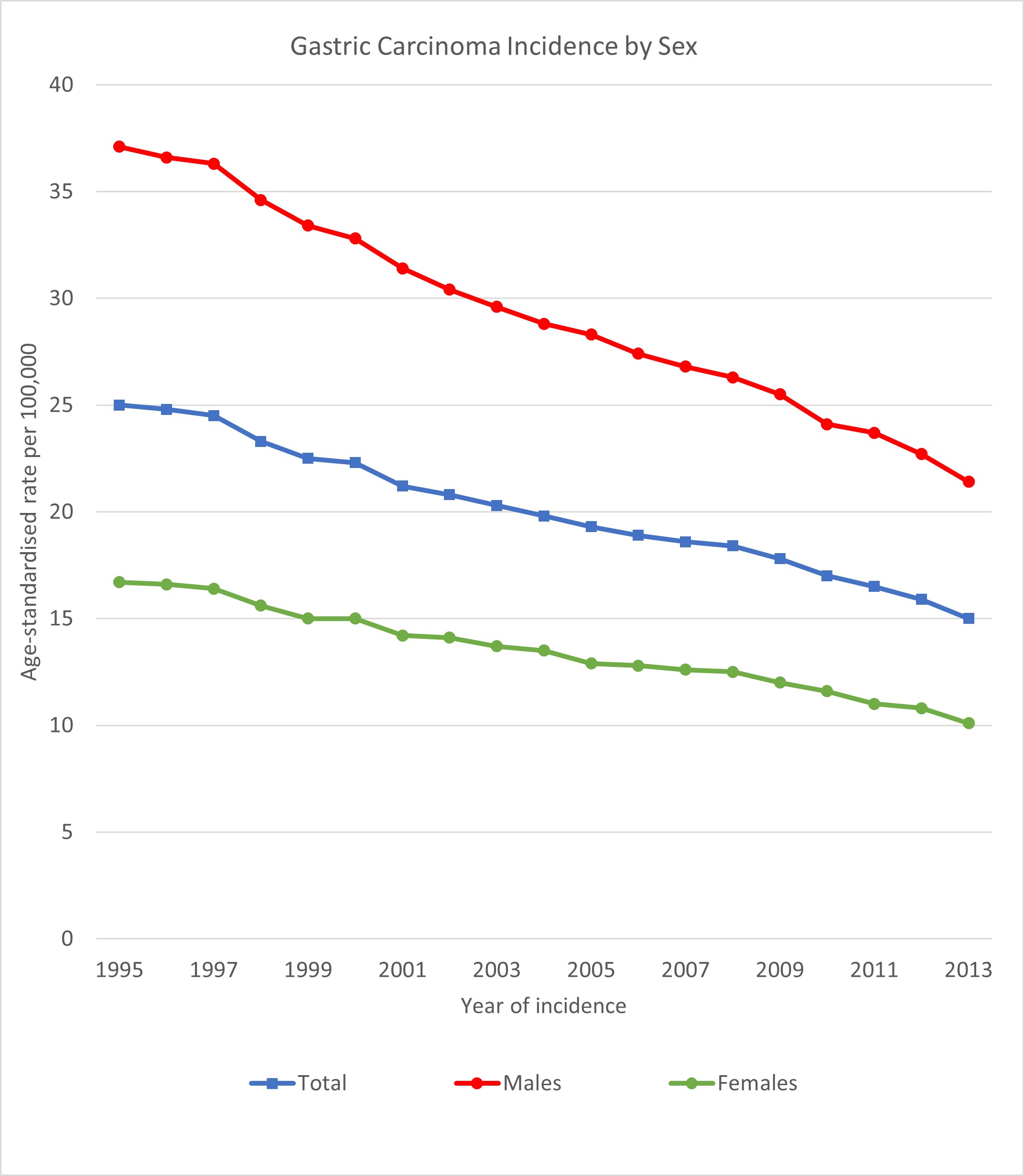

Supplement: Supplementary file 3 [file Image_3.jpeg]

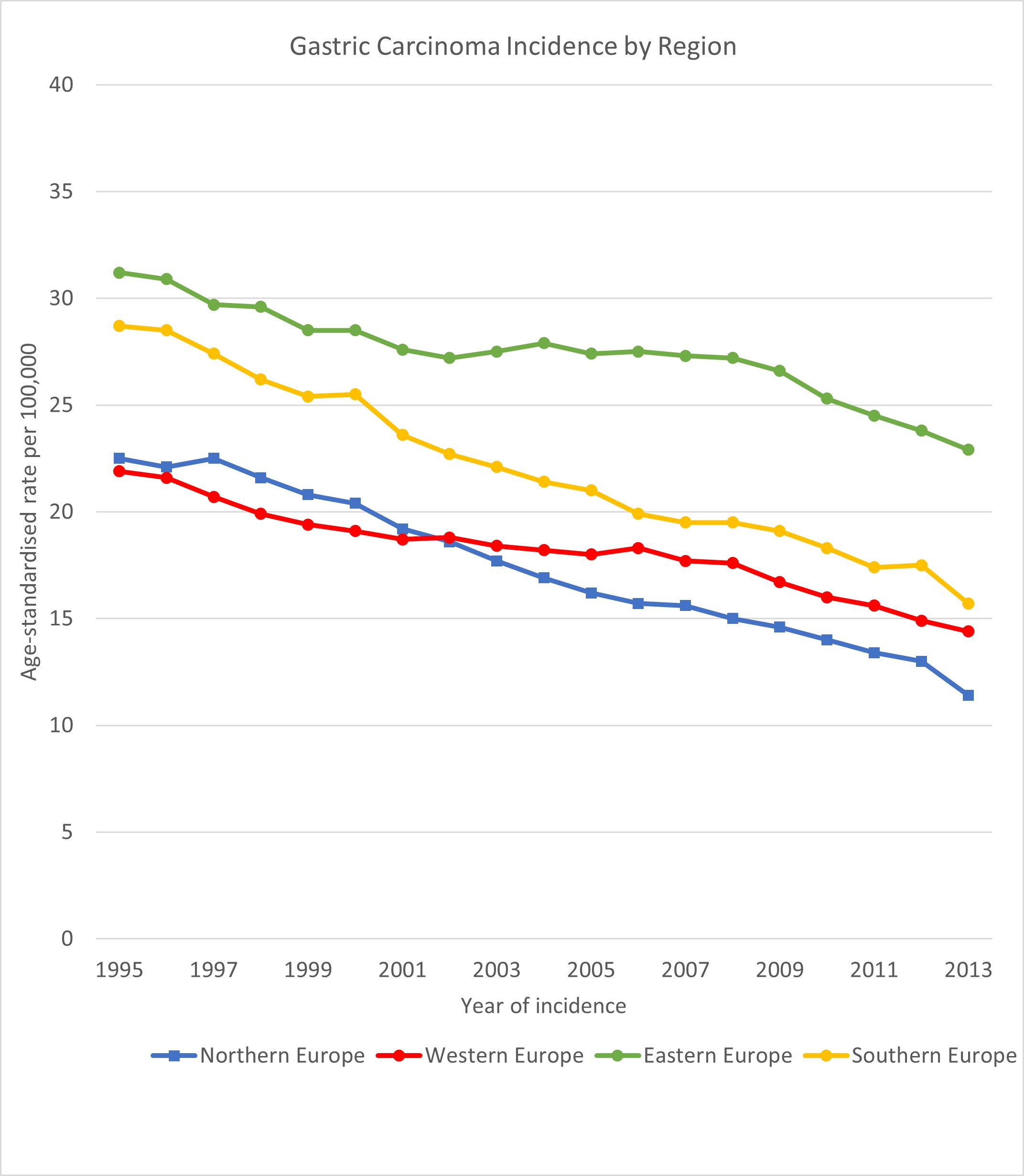

Supplement: Supplementary file 4 [file Image_4.jpeg]
